# Modeling the Role of the Glymphatic Pathway and Cerebral Blood Vessel Properties in Alzheimer’s Disease Pathogenesis

**DOI:** 10.1371/journal.pone.0139574

**Published:** 2015-10-08

**Authors:** Christina Rose Kyrtsos, John S. Baras

**Affiliations:** 1 University of Pittsburgh Medical Center, Department of Neurology, Pittsburgh, Pennsylvania, United States of America; 2 Institute for Systems Research, University of Maryland, College Park, Maryland, United States of America; 3 Department of Electrical and Computer Engineering, University of Maryland, College Park, Maryland, United States of America; Massachusetts General Hospital/Harvard Medical School, UNITED STATES

## Abstract

Alzheimer’s disease (AD) is the most common cause of dementia in the elderly, affecting over 10% population over the age of 65 years. Clinically, AD is described by the symptom set of short term memory loss and cognitive decline, changes in mentation and behavior, and eventually long-term memory deficit as the disease progresses. On imaging studies, significant atrophy with subsequent increase in ventricular volume have been observed. Pathology on post-mortem brain specimens demonstrates the classic findings of increased beta amyloid (Aβ) deposition and the presence of neurofibrillary tangles (NFTs) within affected neurons. Neuroinflammation, dysregulation of blood-brain barrier transport and clearance, deposition of Aβ in cerebral blood vessels, vascular risk factors such as atherosclerosis and diabetes, and the presence of the apolipoprotein E4 allele have all been identified as playing possible roles in AD pathogenesis. Recent research has demonstrated the importance of the glymphatic system in the clearance of Aβ from the brain via the perivascular space surrounding cerebral blood vessels. Given the variety of hypotheses that have been proposed for AD pathogenesis, an interconnected, multilayer model offers a unique opportunity to combine these ideas into a single unifying model. Results of this model demonstrate the importance of vessel stiffness and heart rate in maintaining adequate clearance of Aβ from the brain.

## Introduction

Alzheimer’s disease (AD) is a multi-faceted, progressive neurodegenerative disease. It is the most common cause of dementia in the elderly, affecting nearly 1 in 8 individuals over the age of 65 in the United States [1]. AD has been clinically described by the symptoms of short-term memory deficits, cognitive decline and eventual long-term memory deficit. These deficits lead to a decreased quality of life, often eventually requiring assistance with activities of daily living (ADLs) in a long term care setting [1–2]. There is a tremendous burden for the cost of care even though no preventative or curative treatments are available. Given the increasing number of the elderly affected by AD and the lack of any viable treatment options, there is a pressing need to develop a better understanding of the disease process as well as effective treatment modalities.

Beta amyloid levels within the brain, particularly the oligomeric form, are linked to the loss of neurons, stimulation of neuroinflammation, abnormal mitochondrial function and increase in tau levels [3–10]. Decreased clearance of Aβ from the brain via the glymphatic system and the low-density lipoprotein-related receptor 1 (LRP-1) have been implicated in the pathogenesis of AD, and have been known to occur in normal aging as well [11–12]. The glymphatic system (located in the perivascular space) is believed to drain approximately 60% of the brain Aβ to cervical lymph nodes using convective flow generated by arterial pulsations [13–16]. Increased vessel stiffness causing decreased flow of brain interstitial fluid (ISF) is believed to lead to decreased Aβ clearance with aging [14, 17]. As Aβ concentration builds within the perivascular space, deposition occurs slowly, eventually leading to the breakdown of the blood-brain barrier secondary to degeneration of smooth muscle cells and brain endothelial cells [14]. Beta amyloid deposition in blood vessels has also been shown to inhibit angiogenesis, impair vascular tone and decrease local cerebral perfusion [14, 18]. Clearance of Aβ by LRP-1 has also been shown to decrease by nearly 55% during aging and is believed to be secondary to loss of brain endothelial cells [19].

Given the complex nature of cellular and molecular interactions, mathematical modeling offers a unique opportunity to further understand the pathogenesis of AD. Several other mathematical models of AD have been proposed, with the majority focusing on Aβ fibril nucleation and growth of Aβ plaques [20–21]. Puri and Li developed a network model to study the interactions between neurons, astrocytes and microglia, while Luca et al presented a model on microglia chemotaxis towards Aβ [22–23]. However, there has been no model developed to study the transport of Aβ from generation at the neuron to clearance at the blood-brain barrier or via the glymphatic (eg. perivascular) pathway.

The model presented in this paper studies how Aβ levels change within the brain parenchyma and vasculature during normal aging while incorporating the role of the glymphatic system. Conservation of mass equations are used to model Aβ levels. Neurons, microglia and brain endothelial cells interacted dynamically and responded to both parameters determined by aging process, as well as with the Aβ levels. The role of increased vessel stiffness secondary to aging, prolonged hypertension and cerebrovasculature deposition of Aβ, as well as alterations in heart rate, were also studied given the correlation between cardiovascular risk factors and AD. The results of this model demonstrate the importance of maintaining healthy cardiovascular function and offer insight into possible preventive or treatment options in the future.

## Methods

### Model Assumptions

**Fig 1:**
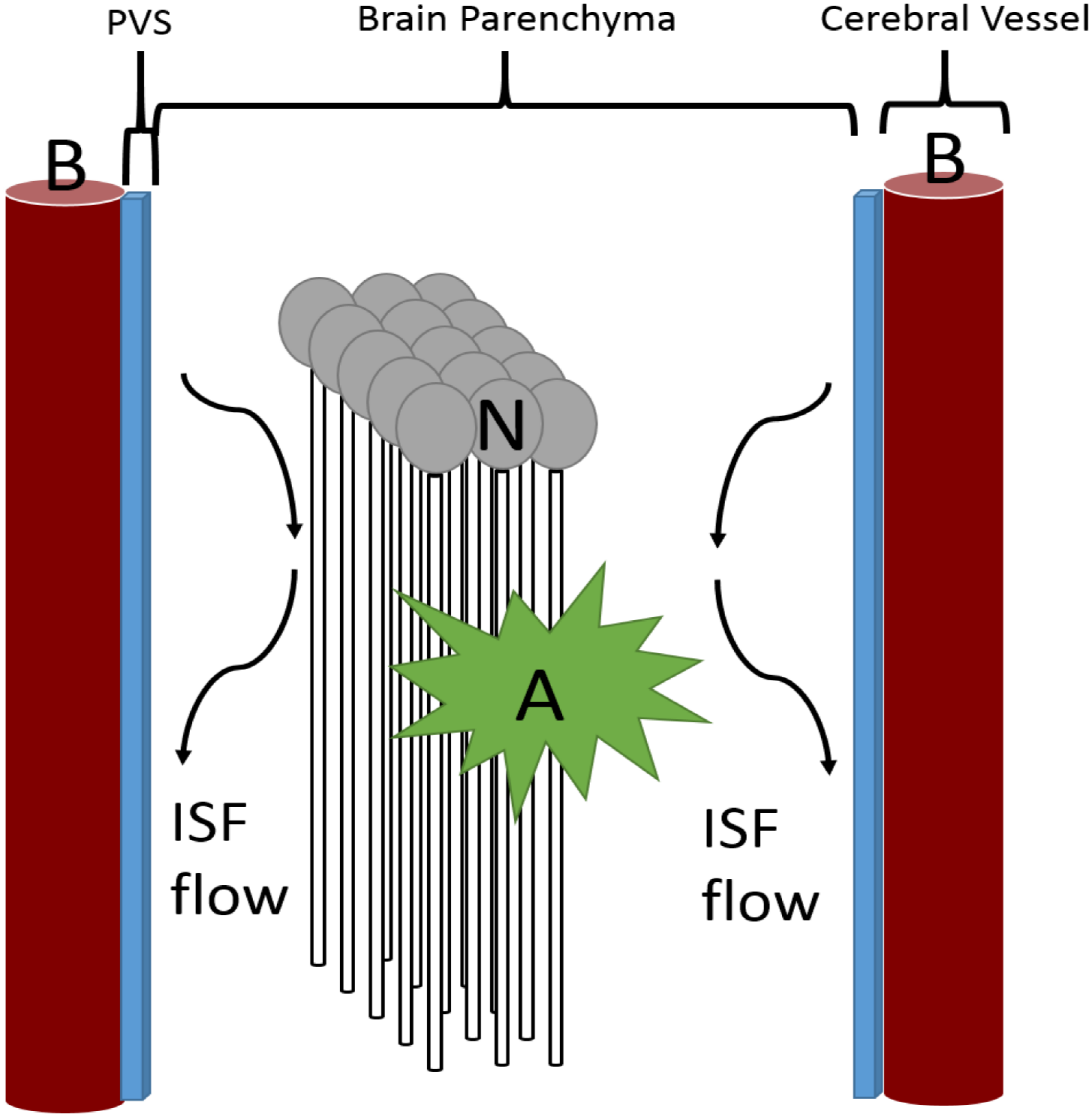
Three compartment model used for modeling. The brain was divided into the brain parenchyma, containing the neurons (N) and astrocytes (A); the perivascular space (PVS, blue strip) in the area between the astrocytic foot processes and the brain endothelial cells; and the cerebral blood vessels (B). Interstitial (ISF) flow is unidirectional and laminar in the regions lined by capillaries; its rate is dependent on heart rate (eg. the rate of arterial pulsations) as well as the stiffness of the vessels, which is a function of the presence of atherosclerosis, cerebral amyloid angiopathy (deposition of beta amyloid within the brain vessels) and stiffening secondary to prolonged elevations in systemic blood pressure.

This model describes the flow of several key chemical species within a 1 mm^3^ volume of CA1 hippocampal tissue (volume of CA1 in healthy patients is approximately 640 mm^3^, total hippocampal volume in healthy patients is around 2100 mm^3^)[24]. The system is divided into three compartments: the blood within the nearby cerebral capillaries; the blood-brain barrier (BBB) and associated perivascular space; and the brain parenchyma, respectively. Capillaries are aligned in parallel with a separation distance of 40 μm [25] and assumed to be have a linear segment length of 1 mm. Neurons and microglia reside within the brain parenchyma between the capillaries and are assumed to have a uniform density. Fig 1 demonstrates the overall layout of the system being modeled.

Each compartment is assumed to be well-mixed with uniform spatial distribution. From recent studies, it has been demonstrated that Aβ is quickly transported from the site of generation via a combination of diffusion (diffusion coefficient, D, range of 1.6–6.23x10^−7^cm^2^/s for monomers to 4x10^−9^ cm^2^/s for oligomers) and convective flow via the bulk brain ISF (0.1–0.3μm/min/g brain tissue [26–28]. Spatial variation of chemical species such as Aβ does occur, however, the time course of this variation given the relatively small intercapillary distance (40 μm [25]) is negligible compared to the time scale used in the simulation.

**Fig 2:**
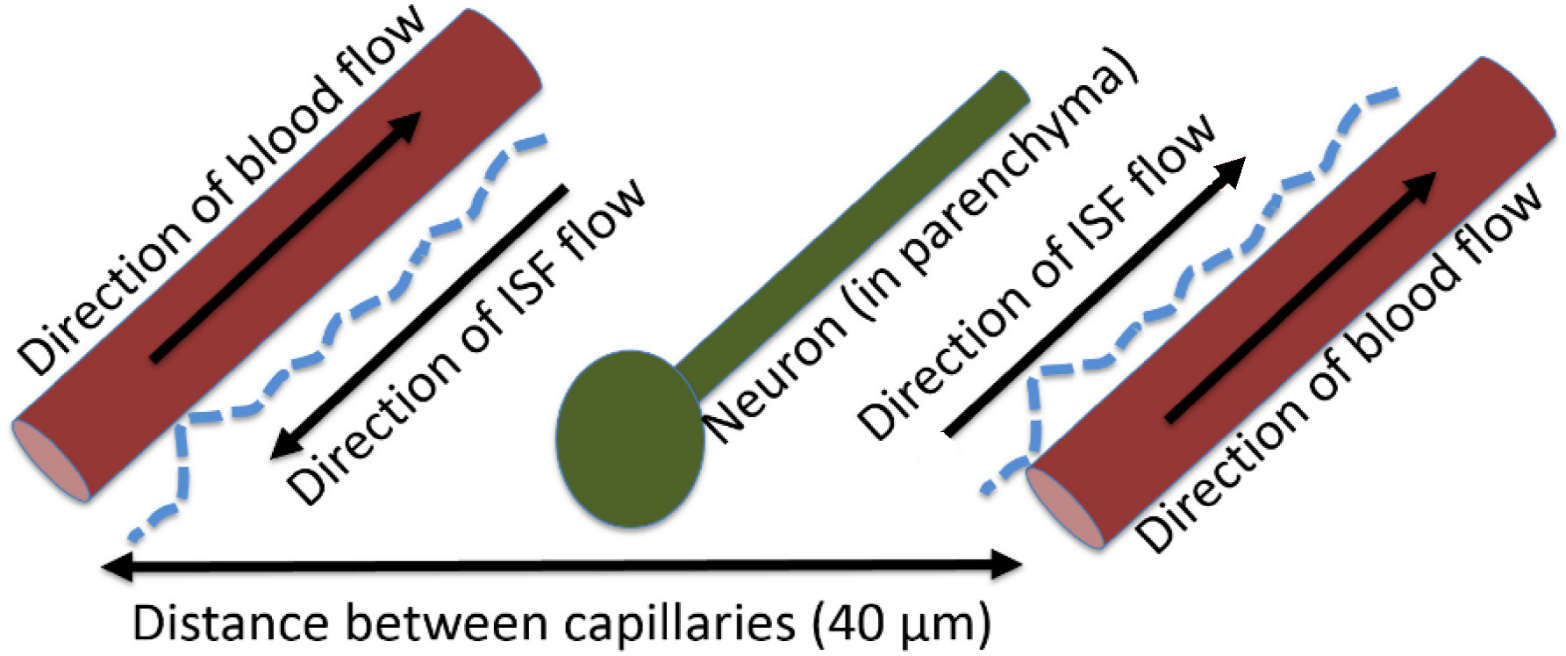
Flow of brain interstitial fluid in response to pulsations of cerebral arteries and arterioles. Interstitial fluid is generated at the brain endothelium by an unclear process [30] and surrounds neurons, microglia astrocytes and other cells located within the brain parenchyma, bringing nutrition to the cells and removing wastes. Although its chemical composition is similar to cerebrospinal fluid (CSF), the two fluids serve separate purposes. CSF gives the brain buoyancy and buffers against forces applied to the head; CSF is generated by ependymal cells within the choroid plexus [30]. Brain ISF transports waste from the brain parenchyma via a combination of convection and diffusion towards the perivascular space. At the perivascular space, molecules such as Aβ, are either transported by receptors at the blood-brain barrier, or are transported along the glymphatic pathway. A small percentage of molecules transported in the perivascular space are transported into the cerebrospinal fluid at the arachnoid granulations and are cleared from the brain via CSF drainage pathways. The majority (~60%) of Aβ is transported along the glymphatic system to the cervical lymph nodes [13].

Beta amyloid is generated by neurons within the brain parenchyma, rapidly mix with brain ISF, and Aβ is transported towards the blood-brain barrier for further transport or clearance [15, 29]. Brain ISF flows along the basement membrane of capillaries and arterioles, to the walls of cortical and leptomeningeal arteries, to the base of the skull before exiting the brain for the cervical lymph nodes [14]. It is not known whether significant levels of transported substances build-up at the cervical lymph nodes. ISF flow is assumed to be unidirectional, well-mixed, and originating from the pulsations of arterial vessels in response to the cardiac cycle (Fig 2). There is some debate in the literature as to whether glymphatic flow is in the same or opposite direction as cerebral blood flow; this model assumes that flow is in the same direction [13, 16]. ISF flow rate is dependent on the heart rate, the ability of the vessel wall to transmit pulsations (eg. the compliance of the arterial wall). Arterial wall compliance can be estimated by wall stiffness (S), which increases with age, hyalinosis secondary to prolonged hypertension, atherosclerosis and vasculitis [14–15]. It is important to note that although brain ISF is generated by brain endothelial cells at the capillary and other vessels, only the arteries and arterioles possess the smooth muscle cells necessary to produce the pulsations to cause ISF flow [30–31].

Once transported to the blood, molecules are rapidly cleared away at a rate of once per second (assuming a heart rate of 60 beats per minute). The blood has also been assumed to be well-mixed and serves as a relative sink for Aβ. Although viscosity of blood can change during aging and disease (eg. diabetes, dehydration), we have not modeled these changes here and assumed that the viscosity and physical properties of blood remain constant throughout the simulation.

### Model Derivation

#### Cell numbers

The volume of the hippocampus has been estimated by both neuropathology and magnetic resonance imaging (MRI) in healthy controls, as well as in patients with mild cognitive impairment (MCI) and AD [24, 32]. Follow-up MRI imaging demonstrated a significant increase in the rate of volume loss in patients with AD or MCI of nearly 5 and 3 times, respectively, the rate of volume loss in healthy controls (table 1) [24]. The total neurons loss in the CA1 region during an average course of AD (15–20 years) has been estimated at 50–60%, much faster than that observed with normal loss during aging (1%, or a 50% loss over 69 years), and demonstrating the impact that the faster loss rate has on the AD-afflicted brain.

**Table 1:** Volume of the human hippocampus as determined by MRI.

Patient	Healthy	MCI	AD
**Baseline volume (mm** ^**3**^ **)**	2133 (25)	1846 (23)	1631 (34)
**Rate of loss/year (mm** ^**3**^ **/yr)**	-17.3 (10.5)	-47.5 (6.5)	-72.0 (9)
**% loss/year (%/yr)**	-0.8 (0.5)	-2.6 (0.3)	-4.4 (0.6)

MCI, mild cognitive impairment; AD, Alzheimer’s disease. Volume loss was approximately 1% in healthy controls, 2.5% in MCI and 4.5% in AD patients. The rate of loss in AD patients was noted to increase by 1.4%/year (total rate of 5.8%.year) in patients with AD carrying the ApoE4 allele. Values are given as the mean with standard deviation in parentheses.

The number of neurons located in the CA1 region of the hippocampus was estimated from the works of Harding and Zarow [33–34]. Zarow et al described the changes in neuron number with respect to the Braak staging, and found that patients with a Braak stage of 0 (essentially healthy, no presence of neurofibrillary tangles) had approximately 3.25x10^6^ CA1 neurons, while those with a Braak score of 6 (severe AD pathology) had only 1.5x10^6^ CA1 neurons [34]. The maximum number of CA1 neurons reported [33] was 6.14x10^6^. Total volume of the CA1 was estimated to be 641 ± 153 mm^3^ in healthy normal patients, decreasing to 377.5 ± 132 mm^3^ in patients with AD [32]. From this, we derived the density of neurons in the CA1 region to be 5275/mm^3^ (4050-6500/mm^3^) in healthy controls and 4500/mm^3^ (range 3000–6000) in AD [17, 35–37]. The change in neuron number over time can thus be described by:

$$\frac{dN}{dt}=\left(1-\left(k_{1}+d\right)\right)N\left(t\right)$$ (1)

where d is the natural rate of neuron loss in the healthy control, k_1_ is the additional neuron loss due to increased beta amyloid levels seen in AD or the presence of the ApoE4 allele, and N is the number of neurons within the control volume.

The density of microglia within several brain regions has been estimated via neuropathology. Tetreault et al found that the average density microglial density in the neocortex is approximately 5951 microglia/mm^3^ [38]. The total number of microglia has been found to vary depending on gender, with women only having 1.9x10^9^ and men having 2x10^9^ microglia [39]. Calculating the microglial density from these values, also noting the slightly lower average brain volume for women, we find that microglial density in women is about 3080 microglia/mm^3^ and 3420 microglia/mm^3^ in men [39]. The number of microglia were also found to decrease over time secondary to dystrophy at a conversion rate of about 80% by 65 years old [40]. Given this range, the microglia density was set at 4500 microglia/mm^3^ with a conversion to dystrophy rate of 7.3x10^−5^/day. The equation for microglia number is given by:

$$\frac{dM}{dt}=\left(1-k_{1_{2}}\right)M\left(t\right)$$ (2)

where k_12_ is the conversion to dystrophy rate and M is the number of microglia within the modeled volume.

Brain endothelial cells (ECs) line cerebral capillaries and work with astrocytes to form the blood-brain barrier (BBB). Between the astrocyte foot processes and the endothelial cells is the perivascular space (PVS), a narrow space (50–100 nm) composed of the basement membrane that allows movement of brain ISF from the capillaries towards the arteries [12, 14, 41]. Endothelial cells in low-flow vessels (such as capillaries) have been found to take on a hexagonal or polygonal shape, with only a single endothelial cell wrapping end-to-end to form the capillary wall. Given that the average capillary wall is approximately 8 μm in diameter, the circumference of the “wrapped” brain capillary endothelial cells can be estimated as 25 μm. Polygonal endothelial cells with this length have been estimated to be between 10–20 μm in width [42–44]. For modeling purposes, we have assumed the final dimensions to be 25 by 20 μm, or approximately 5 endothelial cells per 100 μm capillary length.

Brain ECs have a natural replacement rate of 0.1% per day [45–46], increasing to 1–10% per day if ECs are being exposed to high blood pressure or turbulent flow [45]. During mitosis and EC replacement, some cells are abnormally created leading to EC senescence. This is a process where endothelial cells divide to replace damaged cells and maintain the capillary wall, however, there is local breakdown of the BBB as tight junctions fail to form correctly [47–48]. Denuded spots and disruption of capillary laminar flow have been hypothesized to occur with senescence [47–48]. Approximately 3–7% of ECs are senescent in a 65 year old exposed to normal stressors; this rate increases to 41–93% senescence when ECs are exposed to only twice the level of stressors [47]. Assuming the higher rates of senescence, this translates to a senescence rate of 0.1% per year under normal conditions and 1.4% per year under increased stressors. The equation for the number of brain ECs is then given by:

$$\frac{dEC}{dt}=\left(1-k_{2}\right)EC\left(t\right)$$ (3)

where k_2_ is the rate of ECs entering senescence and EC is the number of brain endothelial cells lining the capillaries in the model volume.

### Chemical Species

#### Beta amyloid

Beta amyloid is generated by neurons after the sequential processing of the 0amyloid precursor protein (APP) by β- and γ-secretase and is found deposited in both the brain parenchyma as well as the cerebral vasculature. The generation rate has been estimated as 3x10^−4^ pg per neuron per day, or the equivalent order of 10^4^ molecules per neuron per day [2]. The ratio of Aβ_42_ to Aβ_40_ generation has been found to be approximately 1:5 [49]. From this data, the generation rate for Aβ_40_ was set at 1.6x10^4^ and Aβ_42_ was set at 3x10^3^. The average concentration of Aβ within brain parenchyma has been estimated using both mice and human brains. Marklund et al used microdialysis probes placed into the brains of patients following traumatic brain injury and noted average Aβ_40_ levels of 436 pg/mL (range 281.3–971.3 pg/mL) and Aβ_42_ levels of 60 pg/mL (range 46–90 pg/mL; values measured in patients with focal brain injury away from the site of injury) [5]. Studies using human brain homogenates have shown similar results (73 pg/mL for Aβ_42_ and 440 pg/mL for Aβ_40_) [35]. Using this range, the initial concentrations for Aβ_40_ and Aβ_42_ are 450 pg/mL and 70 pg/mL, respectively. The general conservation of mass equation can be used to define the change of Aβ within the brain parenchyma as:

$$\frac{\partial C}{\partial t}=\;-\nabla N+R,\;where\;N=\;D\nabla C+vC$$ (4)

where N is the molar flux, D is the diffusion coefficient of Aβ, C is the volume concentration of Aβ, v is the fully developed velocity of the ISF, and R is the volumetric rate of chemical reactions taking place within the control volume given by the rate of Aβ transport by LRP-1 at the BBB. Given the assumptions that flow is unidirectional, fully developed and laminar (given the small length scales modeled) in the region modeled, this equation can be further simplified as:

$$\frac{dC}{dt}=\;-v\frac{dC}{dx}-R$$ (5)

where R represents the generation of Aβ (k_3_, k_4_), the rate of uptake by microglia (k_5_), and the rate of deposition within the brain parenchyma (k_7a_). Degradation of Aβ by extracellular enzymes such as neprilysn have been shown to be negligible *in vivo* [19, 50]. The final equation for Aβ within the brain parenchyma is given by:

$$\frac{dC_{1}}{dt}=\;-v\frac{dC}{dx}+k_{3}k_{4}N\left(t\right)-k_{5}M\left(t\right)-k_{7_{a}}C_{1}\left(t\right)$$ (6)

The concentration of Aβ in the perivascular space (C_2_ for Aβ40 and C_5_ for Aβ42) is derived using a similar simplification of Eq (5) where the concentration differential is still between the brain ISF and the perivascular space. The velocity of the bulk ISF flow was assumed to be approximately double that in an older person and was accounted for in the model. The reaction term is described by the transport rate of Aβ by LRP-1 at the BBB (k_6_) and the rate of deposition in the perivascular space (k_7_):

$$\frac{dC_{2}}{dt}=v\frac{dC}{dx}-k_{6}\left(\frac{LRP\left(t\right)}{LRP_{0}}\right)C_{2}\left(t\right)-k_{7}C_{2}\left(t\right)$$ (7)

where LRP_0_ is the initial concentration of LRP-1 receptors on the brain endothelial cells. A similar equation is used to calculate the Aβ_42_ concentration in the perivascular space (C_4_) with rate constant k_7_ replaced with k_9a_. A term for ApoE was not directly included in Eq (7) given the recent evidence that ApoE may not play a significant role in Aβ clearance by LRP-1 and may actually, in fact, be a competitive inhibitor of Aβ transport since both bind to the LRP-1 receptor [15, 51–52]. Further evidence for this possibility was shown by Shibata et al who noted that there was minimal difference in transport rates in wild-type mice versus ApoE knockout mice [19]. Bell et al confirmed this in their studies of Aβ clearance by LRP-1 and demonstrated that the clearance rate of Aβ bound to ApoE was only about 10% the transport rate of Aβ alone [53]. There is an effect of the ApoE allele, however, on the amount of Aβ transported by LRP-1, as it is believed that ApoE4 may actually bind to LRP-1 longer, thus, increasing potential inhibition of the LRP-1 receptor and slowing clearance of Aβ from the brain [52]. Bachmeier et al demonstrated that clearance of LRP-1 decreased by approximately 2.5 times when the ApoE4 allele was compared to the ApoE3 allele [54].

Review of the literature demonstrated a wide range of transport rates for LRP-1, ranging from 0.015/min to 0.03/min of the fraction of Aβ_40_ injected [19, 55–56]. Bell et al measured the transport rate constant in pmol/min/gram of ISF and found the rate to be 0.211 for Aβ_40_ and 0.111 for Aβ_42_ [53]. Using the conversion for density of brain tissue of 0.99g/cm^3^ [57], this would be equivalent to 12x10^7^ molecules/min/mm^3^ brain, which is more than a magnitude of order elevated beyond the steady state concentration that has been measured *in vivo*. Given the disparity and incongruency in the LRP-1 rate constants in the literature, we used the assumption that 40% of Aβ is cleared by LRP, while the remaining Aβ is cleared by the perivascular pathway or is deposited [11]. This rate of clearance decreased with aging as the density of LRP-1 receptors decreased in response to declining numbers of functioning brain endothelial cells.

Deposition of Aβ within the brain parenchyma (C9) occurred at a constant rate over time dependent on the concentration of Aβ:

$$\frac{dC_{9}}{dt}=k_{7_{a}}C_{1}\left(t\right)\;for\;\text{A}\beta_{4_{0}};\;\frac{dC_{1_{0}}}{dt}=k_{9}C_{4}\left(t\right)\;\;\;for\;\text{A}\beta_{4_{2}}$$ (8)

where C_1_ is the concentration of Aβ_40_ within the brain parenchyma and C_4_ is the concentration of Aβ_42_ in brain ISF. Deposition of Aβ within the cerebral vessel similarly occurred at a constant rate related to the Aβ concentration within the perivascular space:

$$\frac{dC_{3}}{dt}=k_{7}C_{2}\left(t\right)\;for\;\text{A}\beta_{4_{0}};\;\frac{dC_{6}}{dt}=k_{9}C_{5}\left(t\right)\;\;for\;\text{A}\beta_{4_{2}}$$ (9)

#### LRP density

The density of the LRP-1 receptor on brain endothelial cells was determined experimentally by Hvidberg et al to be approximately 31 fmol/mm^3^ [58]. In the model, a 1mm2 area would contain an array of 21 by 21 capillaries. The depth of this array would contain 50 brain ECs, thus, the total density of LRP-1 receptors in the model volume (1mm^3^) would be 9.3x10^11^. During normal aging, the total number of LRP-1 receptors in a given volume decreases by about 55% [19]. Assuming that this loss occurs secondary to the loss of brain endothelial cells with, the rate of LRP-1 loss (k_10_) is equal to the rate of loss of endothelial cells (k_2_):

$$\frac{dLRP}{dt}=\left(1-k_{1_{0}}\right)LRP\left(t\right)$$ (10)

The values for each of the rate constants are given in Table 2.

**Table 2:** Rate constants and initial conditions used in modeling.

Variable	Represents	Units	Value	Reference
**N**	# of neurons	#/volume	5275 (4050–6500)	5, 7–8, 11, 20
**M**	# of microglia	#/volume	4500 (3000–6000)	23–24
**EC**	# of brain ECs	#/length	5 per 100μm (capillaries)	9, 12, 18, 26
**C**_**1**_ **/C**_**4**_	Initial Aβ conc. in brain ISF	pg/ml	Aβ40: 450 (240–770) Aβ42: 70 (40–100)	5, 8, 17
**C**_**2**_ **/C**_**5**_	Initial Aβ conc. in PVS	mass/vol	10% C_1i_/C_4i_	Model assumption
**C**_**3**_ **/C**_**6**_	Concentration of Aβ in vessels	mass/vol	0 (initially)	Model assumption
**C**_**9**_ **/C**_**10**_	Conc. of Aβ deposited in brain	mass/vol	0 (initially)	Model assumption
**k**_**1**_	Loss of neurons when stimulated	%/time	3.4%/yr	7–8
**d**	Natural rate of neuron loss	%/time	1%/yr	7–8
**k**_**2**_	Loss of ECs	%/time	0.1%/yr (no stim) 1.7%/yr (stim)	16, 19
**k**_**3**_ **/k**_**8**_	Aβ_40/42_ generation rate	#/neuron/ time	Aβ_40_: 16000 Aβ_42_: 3000	15
**k**_**4**_	Stimulated Aβ generation rate	%/time	1.5	Estimate
**k**_**5**_ **/k**_**11**_	Microglia uptake rate	#/microglia/ time	Aβ40: 1400 Aβ42: 200	Estimated from 3–4
**k**_**6**_	Aβ transport rate constant for LRP	1/time	ApoE2/3: 0.4 ApoE4: 0.12	2, 21
**k**_**7**_ **/k**_**9a**_	Aβ deposition rate in vessels	fraction/day	Aβ40: 0.004 Aβ42: 0.002	6, 21
**k**_**9**_ **/k**_**7a**_	Aβ deposition rate in brain	fraction/day	Aβ40: 0.001 Aβ42: 0.001	6, 21

Values were derived from experimental studies or estimated given available data. We assumed that there was no Aβ deposited in the brain parenchyma or cerebral vessels at the beginning of the simulation.

#### Simulation conditions

The model was simulated using Matlab. Initial conditions assumed that the model described a 30 year old and was run for a total of 50 years. The timestep used was a single day. Thus, a total of 18,250 data points were determined. Sensitivity analysis was completed for rate constants that were estimated (initial concentration in perivascular space, increased generation of Aβ when a stimulus is present).

## Results

**Fig 3:**
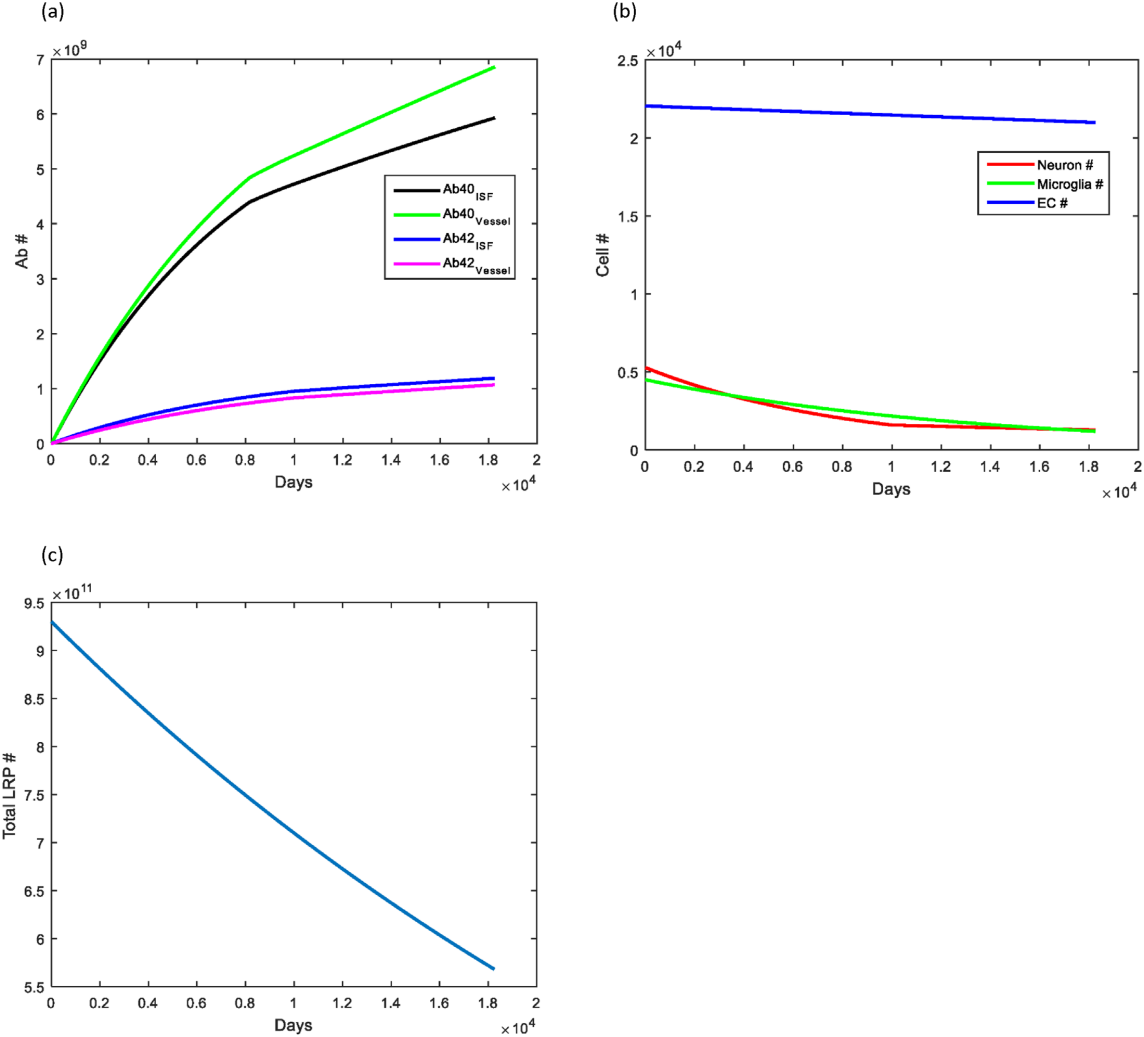
Simulation demonstrating effects of normal aging on Aβ levels within the brain parenchyma and local cerebral vessels. (A) Deposition of Aβ40 occurred much more rapidly than that of Aβ42 with approximately 6 times the amount of Aβ40 deposited within cerebral vessels. This ratio agrees with what has been observed experimentally. The rate of Aβ increase started to decrease over the simulation as the number of neurons declined with aging and from elevations in the Aβ concentration. (B) The number of endothelial cells (blue) decreased slowly as they entered senescence. Microglia (green) decreased about 80% from their initial values, correlating with conversion to dystrophy. Neurons (red) also decreased secondary to natural loss as well as loss from elevated Aβ levels. (C) The total number of LRP-1 receptors decreased to approximately 60% of the initial value in accordance with the loss of brain endothelial cells.

The objective for the model was to study the role that aging has on the clearance of Aβ via the perivascular pathway and transport at the blood-brain barrier by LRP-1. Normal aging causes a slow, steady decrease in the number of neurons in the brain with minor accumulation of Aβ within brain parenchyma and cerebral vessels in the majority of individuals. This was demonstrated clearly in our simulation of normal aging (Fig 3).

**Fig 4:**
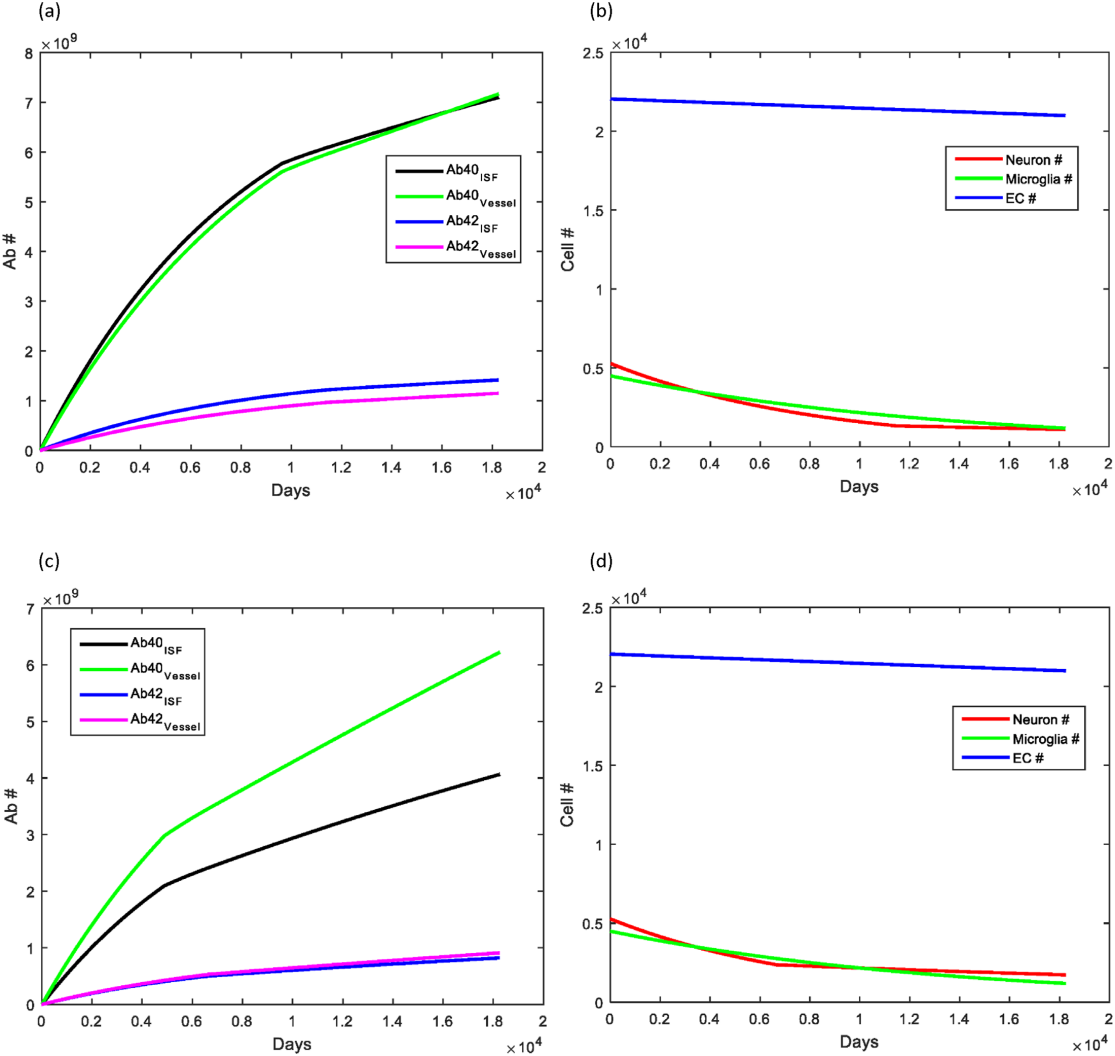
Effect of heart rate on Aβ clearance and deposition. (A, B) Decreasing the heart rate by only 10 beats per minute (60 to 50) led to nearly a 20% increase in the Aβ deposition in the brain parenchyma and a 5% increase in the cerebral vasculature. (C, D) Increasing heart rate by 30 beats per minute (60 to 90) led to the converse, with Aβ deposition levels in brain parenchyma decreasing about 30% and in the cerebral vessels decreasing about 10%.

Heart rate leading to arterial pulsations is important for maintaining the clearance provided by the perivascular glymphatic pathway. During aging, it is common for patients to develop heart rate abnormalities, either too slow of a heart rate (bradycardia), too fast (tachycardia) or an abnormal rate (atrial fibrillation). We hypothesized prior to running simulations that having bradycardia would lead to a decreased Aβ clearance rate and thus, lead to an elevation in Aβ deposition. Conversely, we postulated that an elevated heart rate (for example, from exercising daily) would help to increase the clearance rate and thus decrease the amount of Aβ available for deposition. This was in fact what was seen (Fig 4), with the amount of Aβ deposition in the brain parenchyma increasing by about 20% while deposition within cerebral vessels was minimal (about 5%). Additionally, an elevated heart provided slightly better survival for neuron number.

**Fig 5:**
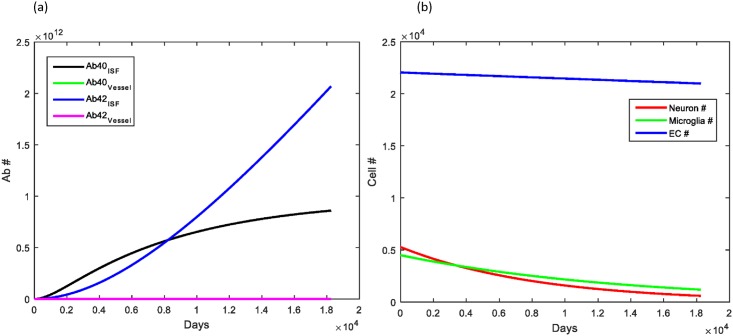
Effect of vessel stiffness on Aβ clearance and deposition. (A) Increasing the stiffness slowly by a factor of 2 during aging led to a significant increase in the amount of Aβ deposited within the brain parenchyma, with a decrease in the amount reaching and thus depositing in cerebral vessels. (B) The high Aβ levels in the brain parenchyma led to accelerated neuronal loss with no change observed in microglia or brain endothelial cell number.

The stiffness of blood vessels is known to increase over time with aging. Vessel stiffness can also increase significantly in diseases such as atherosclerosis and cerebral amyloid angiopathy, two diseases that increase the risk of transitioning to AD in older age. Increasing the stiffness of vessels by a factor of 2 over the course of the simulation via an exponential rise, surprisingly, led to a significant increase in Aβ deposition within brain parenchyma with Aβ_40_ increasing nearly 100x and Aβ_42_ increasing greater than a factor of 10^3^, a level often seen in the brains of those with AD (Fig 5). Beta amyloid deposited within cerebral vessels actually decreased, most likely secondary to less Aβ reaching the perivascular space by convection alone. There was also increased neuronal cell death given the high Aβ levels, with neuron number decreasing to about ½ of the normal age-matched simulation.

**Fig 6:**
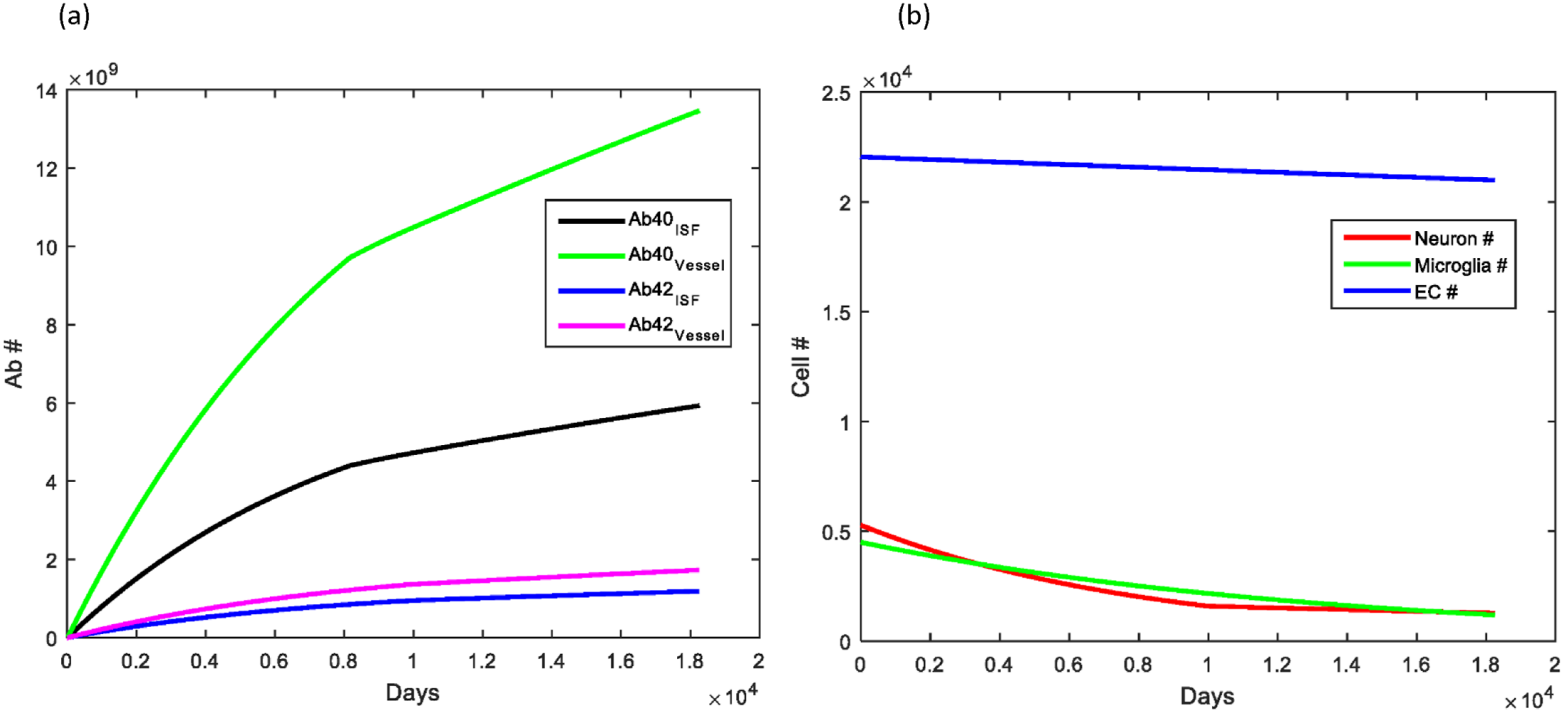
Effect of ApoE allele on Aβ clearance and deposition. (A) Levels of Aβ40 deposition in cerebral vessels increased nearly two-fold with only a 67% increase in Aβ42 deposition there. No changes from baseline simulations were noted in Aβ levels in the brain parenchyma. (B) No changes in cell number were observed compared to baseline values for aging.

The ApoE4 allele is a well-known risk factor for having late-onset AD and is found in 65–80% of all AD cases [59]. Approximately 15–20% of the population is a carrier for ApoE4 (either homozygote or heterozygote), and thus, have an increased risk of AD. The other two alleles (ApoE2 and ApoE3) are not known to contribute to AD risk and are carried by 5–10% and 65–70% of the population, respectively [59]. It is believed that the ApoE4 allele may contribute to elevated Aβ levels by either competitively inhibiting Aβ binding to LRP-1, or by causing inefficient binding of the ApoE*Aβ complex to LRP-1 [51, 59]. Decreasing the transport rate of Aβ by LRP-1 by 2.5 times over the course of the simulation led to approximately a two-fold increase in Aβ_40_ deposition and a 67% increase in Aβ_42_ deposition in cerebral vessels. There was no additional accumulation of Aβ within the brain parenchyma or changes in cell number (Fig 6).

**Fig 7:**
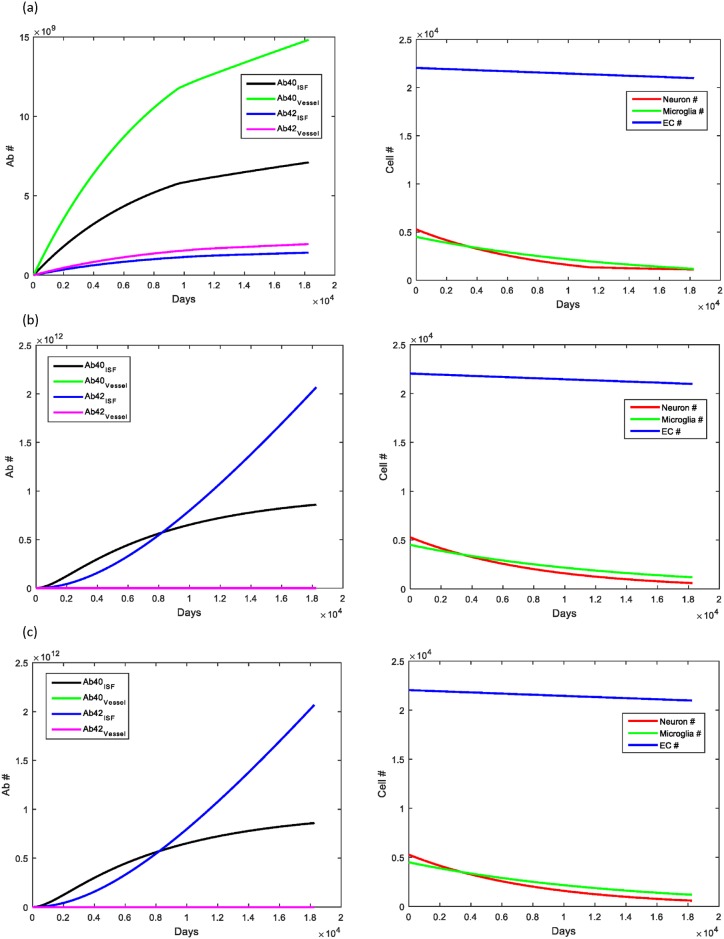
Combination effect of bradycardia, ApoE4 allele and increased vessel stiffness. (A) Having both bradycardia and the ApoE4 allele led to elevated levels of Aβ40 deposition in both the brain parenchyma and the cerebral vessels, with nearly a two-fold increase in Aβ40 in the cerebral vessels. There was a small decrease in the number of neurons at the final simulation endpoint (<10%). (B) In the case where the simulated patient was supposed to have cardiovascular disease (represented by increased vessel stiffness) and carry the ApoE4 allele, Aβ deposition was significantly elevated within the brain parenchyma (100x and 1000x for Aβ40 and Aβ42, respectively). The decreased levels of Aβ within the cerebrovasculature was not quite as significant as with increased vessel stiffness alone, suggesting that the ApoE allele plays an important role in determining deposition and Aβ concentration within the space near cerebral vessels. The significantly elevated Aβ levels led to a decrease in the neuronal cell number secondary to increased neuronal death rate seen at high Aβ levels. (C) The results of increased vessel stiffness in the presence of bradycardia led to results that were similar to those seen with increased vessel stiffness alone.

The final simulations studied the effect of multiple risk factors on changes in Aβ levels. Of the risk factors studied, heart rate and vessel stiffness were the two that could be somewhat modifiable. In Fig 7a, we show that the combination of the ApoE4 allele with bradycardia led to an increase in all Aβ parameters measured, with the most significant increase in Aβ_40_ in cerebral vessels. There was a minimal decrease of less than 10% in the number of neurons. In Fig 7b, we studied the effect of having both the ApoE4 allele as well as a two-fold increase in vessel stiffness. This combination led to a significant increase in Aβ deposition within the brain parenchyma of both forms (Aβ_42_> Aβ_40_). Beta amyloid deposition within the cerebral vessels was decreased compared to normal aging, however, this decrease was not as severe as that seen in with only increased stiffness. This suggests that ApoE has more of an effect on the deposition of Aβ within the cerebral vessels rather than the brain parenchyma. There was significant neuronal loss in this simulation, with no changes noted in the microglia or endothelial cell number. Fig 7c shows the effect of increased vessel stiffness with bradycardia. The results of this simulation were nearly identical to those of increased stiffness alone, suggesting that the effect of bradycardia when there is underlying cardiovascular dysfunction (increased vessel stiffness) is minimal. A summary of the results for each simulation are included in Table 3.

**Table 3:** Summary of simulation results.

Aβ in:	Brain parenchyma	Cerebral Vessel
Normal aging	5.929x10^9^	6.8575x10^9^
	1.187x10^9^	1.0685x10^9^
Bradycardia (HR = 50)	1.20	1.05
	1.19	1.07
Elevated HR (HR = 90)	68.5	90.7
	69.2	85.1
Elevated vessel stiffness (2x)	144.92	9.04x10^−6^
	1741.45	9.07x10^−6^
ApoE4 allele	No change	1.96
	No change	1.62
ApoE4 allele & bradycardia (HR = 50)	1.20	2.16
	1.19	1.83
ApoE4 allele & increased vessel stiffness	144.92	2.9x10^−5^
	1741.45	2.98x10^−4^
Bradycardia & increased vessel stiffness	144.92	9.04x10^−6^
	1741.45	9.07x10^−6^
Increased beta amyloid generation (2x)	1.31	1.29
	1.30	1.30

Under normal aging, Aβ_40_ within the brain parenchyma was found to be 5.929x10^9^, while Aβ_42_ in the brain parenchyma was 1.187x10^9^. Within the cerebral vessel, Aβ_40_ was 6.8575x10^9^ and Aβ_42_ was 1.068x10^9^. The final endpoint value for each simulation was determined and then normalized to the corresponding value obtained for the normal aging simulation to determine the normalized ratio. The normalized ratios for Aβ_40_ are listed first in each row, followed by the value for Aβ_42_. The results here show the dramatic increase in beta amyloid within the brain parenchyma when vessel stiffness increases slowly during aging to a maximum two-fold increase. There is also a corresponding decrease in the amount of Aβ at the cerebral vessel. The only simulation condition that showed a decrease in Aβ was the elevated heart rate to 90 beats per minute. This simulation was trying to model the effect of daily exercise on Aβ clearance and demonstrated the efficacy of exercise in helping to increase Aβ clearance. The final row describes the Aβ normalized ratio when Aβ generation is increased two-fold as part of the sensitivity analysis. This simulation demonstrated that increasing Aβ generation rate had an effect similar to bradycardia in the overall levels of Aβ within the brain parenchyma, but led to further elevations in Aβ within the cerebral vasculature with respect to the bradycardia and normal aging simulations. These results suggests that increased Aβ generation alone is not responsible for the elevated levels of Aβ seen in the brains of patients with AD, though the role of vessel stiffness may play a very important role.

**Fig 8:**
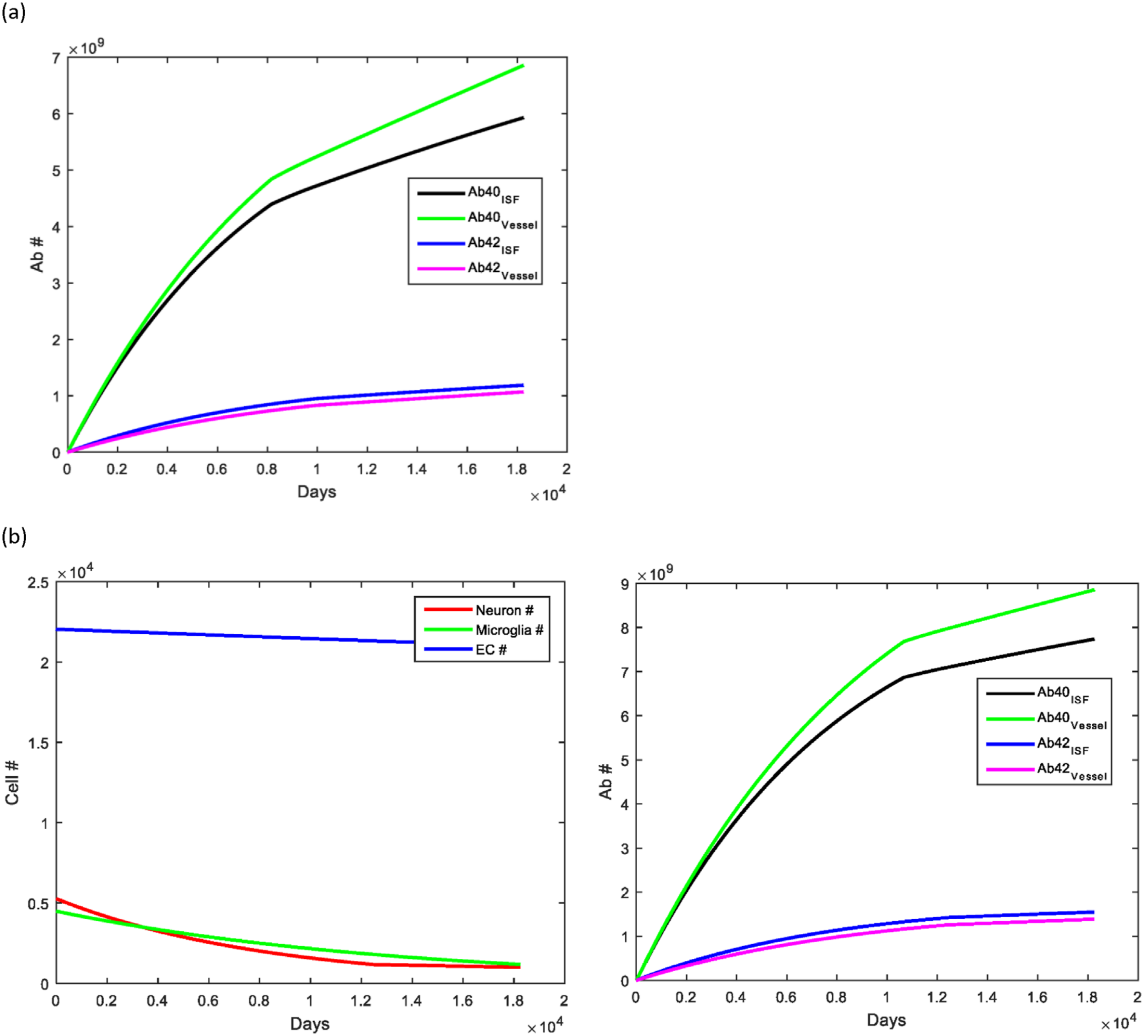
Sensitivity analysis of the model. (A)The sensitivity of the model to values that were estimated was studied to determine the range over which the model produced stable simulation results. Changing the initial concentration of Aβ from ½ x to 2x led to no changes in simulation result and demonstrating stability of this rate constant. (B) Changing the amount that Aβ generation would increase in response to a stimulus (k_4_) showed that this parameter had a relatively narrow range of stability.

A sensitivity analysis was performed on the parameters that were estimated instead of derived from the literature (Fig 8). Changes in the initial concentration of beta amyloid within the perivascular space (C_2_/C_5_) were studied from a range of ½ normal to 2 times normal with no changes in the simulation results observed, suggesting that the model is stable for changes in this value. Analysis on the stability of the k_4_ rate constant (the increase in beta amyloid generation in response to stimuli such as elevated levels of Aβ) demonstrated a narrow range of stability. Increasing k_4_ to 2 from 1.5 led to almost a 30% increase in Aβ levels in all compartments modeled. Further increases in k_4_ (not shown) continued to show a similar trend.

## Discussion

The mathematical model developed in this paper studies the role that clearance by the glymphatic system and LRP-1 at the blood-brain barrier plays in pathogenesis of AD. Altered clearance levels have been implicated as causal factors in the development of AD [60–64]. Results of the model for normal aging demonstrated a gradual increase in Aβ deposition in both the brain parenchyma and the cerebral vessels which correlates well to experimental results [5, 35, 49]. Monitoring neuron, microglia and brain endothelial cell numbers allowed us to track the steady decline in functional cells of each type that occurs normally with aging. One of the limitations of this model is that transport of Aβ by diffusion down its concentration gradient from neurons to the BBB was not incorporated as little experimental data is available at this time. Given what is known of transport properties in the brain, diffusion has been theorized to be an important contributor to movement of chemical species in the brain ISF when species are traveling short distances in the grey matter (few-tens of microns), however, it is believed that convective flow dominates in white matter tracts [26, 65]. Future experimental work will help to better elucidate this mechanism and allow for more accurate modeling.

Cardiovascular risk factors such as the ApoE4 allele, prolonged hypertension leading to re-modeling of vessels and hyperlipidemia have also been recognized as risk factors for AD. In this model, we found that increasing the vessel stiffness by a factor of just 2 from baseline led to a significant increase in the deposition of Aβ in the brain parenchyma most likely secondary to decreased transfer of arterial pulsations and thus, decreased bulk ISF flow. These simulations clearly demonstrated the importance that changes in vessel structure can have in the clearance of Aβ from the brain and offer insight into the disease process, as well as a possible modifiable risk factor. Further research looking at the effect of better blood pressure management and prevention of vessel re-modeling and its effect on AD pathogenesis would provide the necessary evidence to determine if this is a viable AD prevention strategy.

Increasing vessel stiffness in combination with having the ApoE4 allele led to a 10-fold increase in Aβ within the cerebral vasculature compared to the value for increased stiffness only. A similar trend for increased cerebrovasculature deposition of Aβ was seen in other simulations where the ApoE4 allele was included, suggesting the importance that ApoE4 plays in deposition of Aβ in the vasculature. Although ApoE4 is not a modifiable risk factor for AD, this model suggests that being a carrier for it may increase ones risk of having cerebrovasculature deposits of Aβ otherwise known as cerebral amyloid angiopathy (CAA). Further study is still needed to better understand the role that ApoE may play in competitive inhibition of Aβ transport at the blood-brain barrier and its possible role in CAA.

Studying the effect of elevated heart rate on Aβ clearance demonstrated that raising heart rate (90 beats per minute) as can happen during exercise, is beneficial for removing Aβ from the brain. Though this effect was modest, it still demonstrated the positive benefit that exercise can have, especially for the elderly [66–68].

This model represents an attempt to study multi-level interactions between cells within the brain, Aβ levels, LRP-1 levels and the ApoE allele. Future models should look at the interaction between this system and the role that inflammation and cholesterol processing may play in AD pathogenesis. Sleep is also known to be a major determinant of beta amyloid clearance via the glymphatic system, however, experimental rate constants in humans on how shortened lengths of sleep may affect clearance requires further investigation but would be an important parameter to include in future models [69]. Also, as further *in vivo* research that accurately measures parameters for diffusion and convection in the brain become available, including the role of the aquaporin-4 receptor in the glymphatic clearance pathway, the model can be refined to include these newer parameters to more accurately model the system and increase our understanding of divergence of AD progression from normal aging [11, 70].
